# Limited Effectiveness of *Penicillium camemberti* in Preventing the Invasion of Contaminating Molds in Camembert Cheese

**DOI:** 10.3390/foods13182865

**Published:** 2024-09-10

**Authors:** Nicole Ollinger, Alexandra Malachová, Alexandra Schamann, Michael Sulyok, Rudolf Krska, Julian Weghuber

**Affiliations:** 1 FFoQSI—Austrian Competence Centre for Feed and Food Quality, Safety & Innovation, Stelzhamerstr. 23, 4600 Wels, Austria; 2 FFoQSI—Austrian Competence Centre for Feed and Food Quality, Safety & Innovation, Konrad Lorenz Str. 20, 3430 Tulln, Austria; alexandra.malachova@ffoqsi.at (A.M.); alexandra.schamann@ffoqsi.at (A.S.); rudolf.krska@boku.ac.at (R.K.); 3 Department for Agrobiotechnology (IFA-Tulln), Institute of Bioanalytics and Agro-Metabolomics, University of Natural Resources and Life Sciences, Vienna (BOKU), Konrad Lorenz Str. 20, 3430 Tulln, Austria; michael.sulyok@boku.ac.at; 4 Institute for Global Food Security, School of Biological Sciences, Queen’s University Belfast, University Road, Belfast BT7 1NN, UK; 5 Center of Excellence Food Technology and Nutrition, University of Applied Sciences Upper Austria, Stelzhamerstrasse 23, 4600 Wels, Austria

**Keywords:** *Penicillium camemberti*, cyclopiazonic acid, mycotoxins, metabolite, food contamination

## Abstract

Mold-ripened cheese acquires a distinctive aroma and texture from mold cultures that mature on a fresh cheese wheel. Owing to its high moisture content (a_w_ = 0.95) and pliability, soft cheese is prone to contamination. Many contaminating mold species are unable to grow at colder temperatures, and the lactic acid produced by the cheese bacteria inhibits further infiltration. Thus, Camembert cheese is generally well protected against contamination by a wide range of species. In this study, cocultures of *Penicillium camemberti* and widely distributed mycotoxin-producing mold species were incubated on different types of agars, and purchased Camembert samples were deliberately contaminated with mycotoxin-producing mold species capable of growing at both 25 °C and 4 °C. The production of mycotoxins was then monitored by the extraction of the metabolites and their subsequent measurement by means of a liquid chromatography-tandem mass spectrometry (LC-MS/MS) based targeted metabolite profiling approach. The production of cyclopiazonic acid (CPA) was highly dependent on the species cocultivated with *Penicillium camemberti*, the temperature and the substrate. Contamination of Camembert cheese with *Penicillium chrysogenum*, *Mucor hiemalis*, or *Penicillium glabrum* induced CPA production at 25 °C. Although mold growth on cheese was not always evident on biofilms for certain cultures, except for *Penicillium citrinum*, which stained the monosaccharide agar yellow, mycotoxins were detected in many agar and cheese samples, as in all monosaccharide agar samples. In conclusion, cheese should be immediately discarded upon the first appearance of mold.

## 1. Introduction

Food fermentation is a long-established method of either preserving food, changing its texture or adding certain flavors and tastes. Japanese soy sauce is a seasoning sauce made by fermenting soybeans with lactic acid bacteria, salt-tolerant yeast and koji molds. Galician chorizo is also fermented with *Lactobacillus* and *Staphylococcus.* These starter cultures ensure continuously consistent quality, extended shelf-life, and homogenous production [1,2].

In some products, in addition to influencing flavor and texture, the cultures produce a protective biofilm to prevent the growth of undesired microorganisms [3,4]. These biofilms should mainly protect against larger amounts of bacteria, such as *Escherichia coli* or *Salmonella*, which cause, among other symptoms, diarrhea, nausea, abdominal pain, and vomiting [5]. In many cases, fungal contamination itself is not a problem. However, under certain conditions, fungi produce toxins that have health consequences, and their growth should therefore be prevented. The production of ochratoxin A induced by *Penicillium verrucosum* contamination is reduced to below the detection limit by a protective biofilm of *Penicillium nalgiovense* during the maturation of dry fermented Salchichon sausage [6].

Many dairy products, such as yogurt, kefir, or cheese, are also the result of fermentation. The microbes used for the fermentation of these foods have a low toxigenic capacity. Brie, Camembert, and Roquefort are among the most common types of mold cheese. While ripening cultures are harmless, pathogens can still produce mycotoxins. Fungal secondary metabolites can be detected in dairy products originating from two different sources. First, indirect contamination occurs when dairy cows consume feed containing mycotoxins, which are subsequently transferred to milk, which can include the mycotoxin aflatoxin M_1_ as a metabolite of aflatoxin B_1_. Second, direct contamination is a consequence of the deliberate or accidental proliferation of molds. Among mycotoxins, citrinin, penitrem A, roquefortine C, sterigmatocystin, and aflatoxin have been shown to be stable in cheese. In contrast, mycotoxins such as patulin, penicillic acid, and PR toxin do not persist in this environment [7].

Studies have been carried out on contaminated food samples. In summary, the amount and distribution of mycotoxins in food depend on the availability and concentration of sugars, the water content, the storage temperature, the storage time, and, of course, the strain of mold inoculated. The diffusion of mycotoxins also depends on the liquidity of the food sample. In more liquid foods, such as crème fraiche or jam, toxins can diffuse much faster than they can in hard cheese [8,9].

Moreover, polar or small mycotoxins may diffuse more rapidly. A high sugar content, such as that in jam, can prevent mold growth and therefore mycotoxin formation [8,9].

Psychrophilic fungi, such as *Mucor hiemalis* and *Penicillium chrysogenum*, which can contaminate soft cheeses, are ubiquitous and have even been found on a glacier [10]. Several mold species were screened for ubiquity, low-temperature growth, and mycotoxin production in an internal experiment not yet published. *Mucor hiemalis*, *Penicillium chrysogenum*, and *Penicillium glabrum* were found to present all of these characteristics. Therefore, testing the growth abilities of these contaminating species is highly important. Thus, mycotoxin production in cocultivations of *Penicillium camemberti* and potential contaminating species was tested at 4 °C and 25 °C in the present study. These temperatures were chosen because the common storage temperature for Camembert cheese is 4 °C, whereas 25 °C is the accepted cultivation temperature for *Penicillium*. Thus, the choice of these temperatures allows easy comparison with past and future datasets.

**Figure 1:**
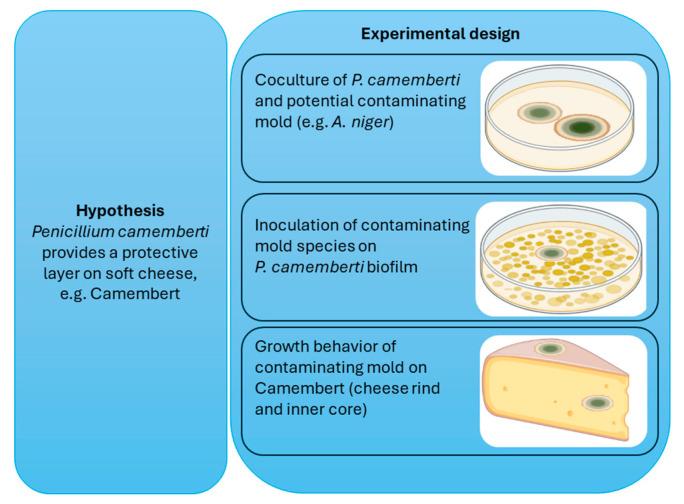
Overview of experimental design. A parallel coculture and a biofilm culture with *Penicillium camemberti* and a potential contaminating mold was prepared to determine mycotoxin production of respective species. Additionally, Camembert cheese was inoculated with mold on the rind and on the inner core.

Although mold cheeses such as Brie, Roquefort, or Camembert are common products, the European Food Safety Authority (EFSA) states that *Penicillium camemberti* is ineligible for a qualified presumption of safety assessment [11]. However, the intake of fermented soft cheese remains high, primarily because of its texture, taste, and scent. In Germany, the production of soft cheese was as high as 165.000 tons in 2022 [12]. A previous study showed that 32% of the 19 tested commercial Camembert samples contained 89–120-µg/kg CPA [13]. Due to the possibility of ingesting mycotoxins and the high demand for fermented cheeses, research is required to ensure the safety of soft cheese consumption. These studies are currently lacking. Therefore, this research is focused on the question of whether the fungal species *Penicillium camemberti* not only provides beneficial taste and flavor to cheese but also serves an additional function of creating a protective film against other microorganisms. The mycotoxin production of pure and cocultivated cultures of *Penicillium camemberti*, *Chaetomium globosum*, *Aspergillus niger*, *Paecilomyces variotii*, *Penicillium citrinum*, *Penicillium chrysogenum*, *Mucor hiemalis*, and *Penicillium glabrum* was tested under different cultivation conditions, such as different substates and different temperatures, after 14 days of incubation. A flowchart on the experimental design is given in Figure 1. The induction of mycotoxin production on inoculated cheese samples at 4 °C and 25 °C was tested as well as the mycotoxin production on potato dextrose agar at 25 °C, was tested with *Penicillium camemberti* and five contaminating fungal species inoculated at a distance of 3 cm. In addition, the mycotoxin production ability of the same contaminants was tested on a *Penicillium camemberti* biofilm growing on two types of sugar agar at 25 °C.

## 2. Materials and Methods

### 2.1. Chemicals, Cultures, and Culture Media

Chemicals and culture media were obtained from Sigma Aldrich (Schnelldorf, Germany) unless otherwise stated. Potato dextrose agar (PDA), malt extract agar, oatmeal agar, and four customized agar mixtures containing (1) 2% starch and 3.6% glucose, (2) 2% starch, (3) 4.8% lactose, or (4) 2.5% glucose and 2% galactose, with 2% agar and potassium phosphate buffer at pH 6.0, and common trace elements and micronutrients were used. The agar media were autoclaved at 121 °C for 20 min and poured warm into 9 cm sterile petri dishes. The media were allowed to polymerize at room temperature for at least 4 h and kept at 4 °C until use.

Lyophilized cultures of *Paecilomyces variotii* (CECT 20360), *Penicillium citrinum* (CECT 2274), *Penicillium chrysogenum* (CECT 2307), *Aspergillus niger* (CECT 2088), and *Penicillium camemberti* (CECT 2267) were purchased from the Spanish Type Culture Collection (CECT, Paterna, Spain). *Chaetomium globosum* (DSM 1962) and *Mucor hiemalis* (DSM 2655) were obtained from the German Collection of Microorganisms and Cell Cultures GmbH (DSMZ, Braunschweig, Germany). *Penicillium glabrum* was isolated from a moldy cream cheese in-house and identified by DNA barcoding. A mesophilic starter culture consisting of a variety of lactic acid bacteria was purchased from MilkySky GmbH (Lauben, Germany).

### 2.2. Water Activity

Water activity (a_w_) was measured in triplicate using a LabMaster-a_w_ device (Novasina AG, Lachen, Switzerland) on agar plates and cheese. Samples were taken from the centers of the cheeses and the centers of the agar plates, as water activity is a critical parameter for fungal growth.

### 2.3. Cultivation on Agar Plates

Cocultivations of contaminating fungal species and *Penicillium camemberti* were conducted in two ways to determine if it made a difference if a biofilm was present at the beginning or if both species started growing at the same time.

For parallel cocultivation, 35,000 spores of each of the contaminating species, *Chaetomium globosum*, *Aspergillus niger*, *Paecilomyces variotii*, *Penicillium citrinum*, and *Penicillium chrysogenum*, were carefully pipetted onto a separate PDA plate. The same number of spores of the test strain *Penicillium camemberti* were pipetted onto the same plate at a distance of 3 cm. The remaining liquid of the spore suspension was allowed to evaporate so that all the spores were located at the same starting spot. After the plates were sealed with parafilm to prevent unwanted contamination, they were incubated at 25 °C for 14 days. The plates were photographed before aliquots were taken for analysis. PDA was chosen because it induced low but measurable mycotoxin production in each of the mold species tested. To quantify the mycotoxin production of pure cultures, each species was inoculated individually onto PDA plates at the same time to ensure comparability. The pure cultures were prepared in duplicate, except for *Penicillium camemberti*, which was prepared in triplicate, and the cocultivation plates were prepared in triplicate.

For biofilm cocultivation, 35,000 spores of *Penicillium camemberti* were spread evenly on all five types of agar plates and incubated at 25 °C. After 2 days, a thin layer had formed, on which 35,000 spores of the contaminating strains were pipetted onto the center of the plate and incubated for 14 days at 25 °C or at 4 °C for the temperature comparisons. The plates were photographed before aliquots were taken for analysis.

To test cultures for psychrophilism and low-temperature resistance, spores of *Penicillium chrysogenum*, *Penicillium citrinum*, *Penicillium camemberti*, *Aspergillus niger*, *Chaetomium globosum*, *Mucor hiemalis*, *Penicillium glabrum*, and *Paecilomyces variotii* were transferred to malt extract and oatmeal agar and incubated at 4 °C for 2 weeks to test their ability to grow at relatively low temperatures in the dark.

### 2.4. Mycotoxin Production Induced by Biofilm Cocultivation of Contaminating Species, Lactic Acid Bacteria, and Penicillium camemberti

*Penicillium camemberti* was spread on lactose and glucose/galactose agar plates and incubated at 25 °C to form a biofilm. After 2 days, *Penicillium chrysogenum*, *Penicillium citrinum*, *Aspergillus niger*, *Chaetomium globosum,* and *Paecilomyces variotii* were spread on the light lawn and incubated for 2 weeks. To observe the growth of the contaminating species, *Aspergillus niger*, *Penicillium chrysogenum,* and *Chaetomium globosum* were inoculated on a lawn of lactic acid bacteria on lactose and glucose/galactose agar.

Approximately 35,000 spores were used per inoculation. After 14 days, photographs and aliquots were taken to determine the mycotoxin load between the mold growth areas.

### 2.5. Biofilm Cocultivation of Penicillium camemberti and the Contaminating Species Mucor hiemalis, Penicillium chrysogenum, and Penicillium glabrum at 4 °C and 25 °C

The abilities of *Mucor hiemalis, Penicillium chrysogenum,* and *Penicillium glabrum* to grow at 4 °C were tested. To test mycotoxin production at 4 °C and 25 °C, a biofilm of *Penicillium camemberti* was spread and incubated for 2 days at 25 °C as previously described. PDA, starch/glucose agar, starch agar, lactose agar, and galactose/glucose agar were used to monitor mycotoxin production in the presence of different carbon sources and compositions. Spores of *Mucor hiemalis*, *Penicillium chrysogenum,* and *Penicillium glabrum* were pipetted into the center of each plate and incubated at 4 °C and 25 °C for an additional 14 days. Aliquots were taken from the center of each plate for mycotoxin analysis.

### 2.6. Cheese Contaminated with Strains of Mucor hiemalis, Penicillium chrysogenum, and Penicillium glabrum

Two loaves of Camembert cheese were purchased from a local supermarket and cut into four quarters using a sterile scalpel. The quarters were then cut horizontally to further expose the inside of the cheese. Spores of *Penicillium chrysogenum*, *Mucor hiemalis*, and *Penicillium glabrum* were inoculated inside each Camembert sample and the rind. Mycotoxin production was determined after the cheese samples were stored at 4 °C and 25 °C.

The initial mycotoxin load was determined by randomly taking three aliquots from fresh cheese. In addition, untreated cheese aliquots were stored at 4 °C and 25 °C to observe changes in initial mycotoxin concentrations due to storage conditions. Samples for analysis were taken from the center of the inoculation location.

### 2.7. Mycotoxin Analysis

The extraction and analysis of fungal secondary metabolites were performed as described previously [14] with slight modifications. Briefly, metabolites were extracted from one agar block using 1 mL of extraction solvent (acetonitrile/water/acetic acid, 79:20:1, *v*/*v*/*v*) by shaking for 90 min on a GFL rotary shaker (Burgwedel, Germany). Subsequently, 500 µL of the supernatants were diluted in High Performance Liquid Chromatography (HPLC) vials with 500 µL of acetonitrile/water/acetic acid (20:79:1, *v*/*v*/*v*). After vortexing, 5 µL of the diluted extract was injected into an LC-MS/MS system. The samples were measured on a 1290 Series Agilent Technologies HPLC System (Waldbronn, Germany) connected to an Applied Biosystems SCIEX QTrap 5500 LC-MS/MS System (Framingham, MA, USA) equipped with a Turbo Ion Spray electrospray ionization source. The separation was achieved at a flow rate of 1 mL/min on a Gemini^®^ C18 column, 150 × 4.6 mm i.d., 5 μm particle size, equipped with a C18 4 × 3 mm i.d. Phenomenex security guard cartridge (Torrance, CA, USA) at 25 °C. The identification of a positive metabolite was confirmed by the acquisition of two MS/MS signals per analyte in the time-scheduled multiple reaction monitoring mode, which generated 4.0 identification points according to the European Commission decision 2002/657. Furthermore, the retention time and ion ratio were compared to the related values of authentic standards, allowing for maximal deviations of 0.03 min and 30% rel., respectively. External calibration using serial dilutions of a multianalyte stock solution was performed to quantify most metabolites. For a few metabolites for which no standard was available, the area under the curve was used for the calculations. The performance of the method was confirmed on a continuous basis by its use in a proficiency testing scheme, with more than 96% of the 2200 results submitted thus far exhibiting a z score of −2 < z < 2.

## 3. Results

An overview of the experimental design is shown in Figure 1.

### 3.1. Parallel Cocultivation of Contaminating Species and Penicillium camemberti

The species *Chaetomium globosum*, *Aspergillus niger*, *Paecilomyces variotii*, *Penicillium citrinum*, and *Penicillium chrysogenum* were incubated on PDA for 14 days at 25 °C at a distance of 3 cm from *Penicillium camemberti*.

**Figure 2:**
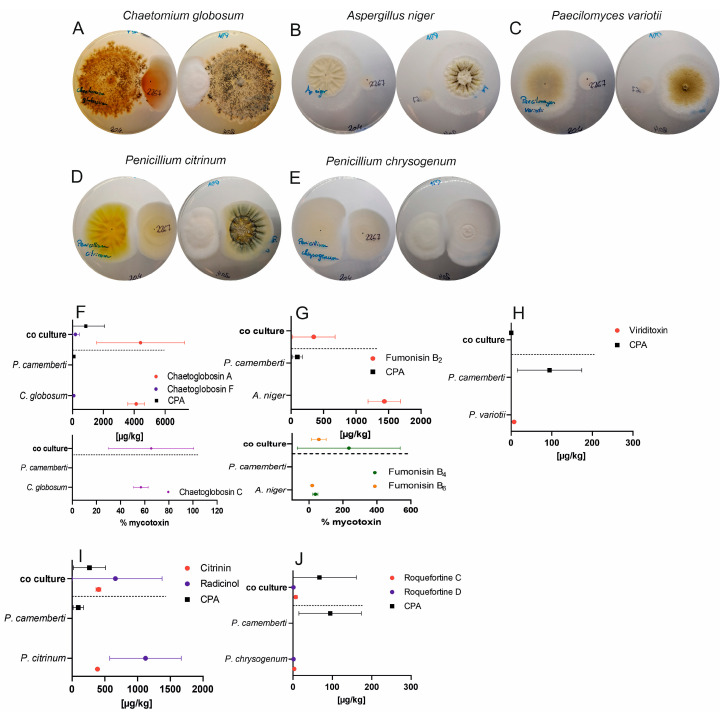
Images from the top and bottom of parallel cocultures of five contaminating fungal species incubated 3 cm away from *Penicillium camemberti* on PDA for 14 days (**A**–**E**) and mycotoxin production of the strains as pure cultures as well as in coculture (**F**–**J**). As no standards are available for chaetoglobosin C, fumonisin B_4,_ or fumonisin B_6_, the levels of these compounds in the cocultures are expressed as relative % changes compared with the levels in the pure cultures by setting one value of the measurement equal to 100% and calculating the remaining values (**F**,**G**). The LOD of fumonisin B_2_ was 2.7 µg/kg (*n* ≥ 2).

The growth behaviors of five mold strains in the presence of *Penicillium camemberti* were documented in photographs, as shown in Figure 2A–E. Fungal mycotoxin production is shown in Figure 2F–J and is summarized in Table 1.

*Chaetomium globosum* overgrew *Penicillium camemberti* (Figure 2A). Mycotoxins formed by *Chaetomium globosum*, such as chaetoglobosin F, tended to increase during coculture compared with those in pure cultures. Cyclopiazonic acid (CPA) production by *Penicillium camemberti* tended to increase strongly (Figure 2F).

*Aspergillus niger* overgrew *Penicillium camemberti* (Figure 2B), and compared with pure cultures, the levels of fumonisin B_4_ and fumonisin B_6_ were increased in cocultures, whereas the fumonisin B_2_ level was lower, and hardly any CPA was detected (Figure 2G).

The cocultivation of *Paecilomyces variotii* and *Penicillium camemberti* revealed that the strains tried to avoid contact with each other (Figure 2C), but both stopped the production of mycotoxins in parallel cocultures (Figure 2H).

Visual inspection of the *Penicillium citrinum* coculture (Figure 2D) revealed the same avoidance situation, but mycotoxin production remained almost the same, except for the CPA level, which tended to increase (Figure 2I).

*Penicillium chrysogenum* and *Penicillium camemberti* also did not favor each other (Figure 2E). CPA production by *Penicillium camemberti* decreased, the production of roquefortine C by *Penicillium chrysogenum* doubled, and the roquefortine D concentration tended to decrease slightly compared with those of the pure cultures.

### 3.2. Cocultivation of Penicillium camemberti Biofilms

The parallel inoculated cultures, which were started with the same number of spores and at the same time, revealed that there was no clear dominant behavior of one of the contaminating strains or of *Penicillium camemberti*. Under cheese production conditions, contamination usually occurs at much lower spore concentrations than that of the desired fungus. Therefore, we inoculated *Penicillium camemberti* on customized lactose agar and monosaccharide agar to determine the mycotoxin production when *Penicillium camemberti* was predominant. Mycotoxin production in the presence of lactic acid bacteria was also determined. Again, 35,000 spores of *Penicillium camemberti* were spread evenly on the plates and incubated for 2 days at 25 °C to allow the fungus to grow. Then, 35,000 contaminated spores were pipetted into the center of the plate. Lactic acid bacteria used in cheese production were added to other plates to ensure the growth of a dense lawn, and after 10 min of drying, the *Penicillium camemberti* spores were added. All the plates were subsequently incubated for 14 days at 25 °C.

**Figure 3:**
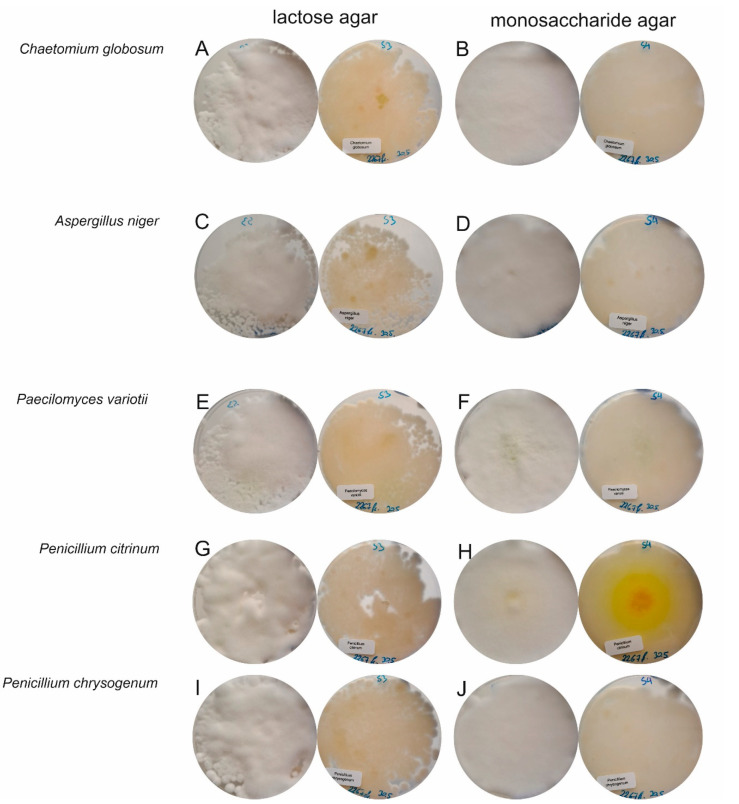
Fourteen days of biofilm cultivation of *Penicillium camemberti* and five contaminating species, namely, *Chaetomium globosum*, *Aspergillus niger*, *Paecilomyces variotii*, *Penicillium citrinum*, and *Penicillium chrysogenum*, on two types of customized agar with either lactose or monosaccharides as the carbon source. The top view is on the left-hand side of each panel, and the bottom view is on the right-hand side of each panel. (**A**,**B**) *Chaetomium globosum*, (**C**,**D**) *Aspergillus niger*, (**E**,**F**) *Paecilomyces variotii*, (**G**,**H**) *Penicillium citrinum*, (**I**,**J**) *Penicillium chrysogenum*.

Figure 3 shows the biofilm cocultivation plates. The contamination was not visible except for *Penicillium citrinum*, whose yellow metabolites were visible at the bottom of the monosaccharide agar. The corresponding mycotoxin concentrations are summarized in Table 2. The production of CPA, the mycotoxin formed by *Penicillium camemberti,* was induced only by *Penicillium citrinum* and lactic acid bacteria on lactose agar, and a high level of CPA (1000 µg/kg) was measured. *Penicillium citrinum* and *Paecilomyces variotii* produced low amounts of the metabolites citrinin and viriditoxin, respectively, on lactose agar. In contrast, the production of CPA was detectable in each biofilm cocultivation plate containing monosaccharides, except for the pure *Penicillium camemberti* plate. *Penicillium citrinum* produced approximately 400 µg/kg citrinin and approximately 1810 µg/kg radicinol, and *Paecilomyces variotii* produced approximately 170 µg/kg viriditoxin. The a_w_ values of both agars were similar at 0.95 for lactose agar and 0.94 for galactose/glucose agar and therefore had no effect.

### 3.3. Mycotoxin Production Is Increased in Biofilm Cocultures at 25 °C Compared with Those at 4 °C

Although the induction of mycotoxin production was not as excessive as expected, the question arose as to whether mycotoxin production could be reduced when samples were stored at 4 °C. Most of the strains evaluated previously did not grow at 4 °C, so the nongrowing test strains were replaced, and proliferation and mycotoxin production were tested with *Penicillium chrysogenum*, *Mucor hiemalis*, *Penicillium camemberti*, and *Penicillium glabrum*. To ensure a greater diversity of carbon sources in the substrates, five customized types of agars were used in addition to PDA (Table 3).

The four fungal strains were incubated on five agar plates at 25 °C to obtain reference values in pure cultures. The mycotoxin levels were below the limits of detection on all plates, except for the following: *Penicillium chrysogenum* and *Penicillium camemberti* cocultures produced approximately 140 µg/kg roquefortine C and 10 µg/kg roquefortine D on PDA. CPA was produced on all the coculture plates, at least on PDA. CPA was measured at a concentration of 7090 µg/kg at 25 °C in samples taken from cocultures of *Penicillium chrysogenum* and *Penicillium camemberti* incubated on PDA, but no CPA was detected in samples incubated at 4 °C. The same situation was observed on starch/glucose agar (60 µg/kg) and starch agar (540 µg/kg), while the starch agar samples contained a small amount of CPA (40 µg/kg) at 4 °C. On lactose agar and galactose/glucose agar, a small amount of CPA was detected only in samples incubated at 4 °C.

In biofilm cocultures with *Mucor hiemalis*, the CPA concentrations were 2460 µg/kg on PDA incubated at 25 °C and 10 µg/kg after incubation at 4 °C, whereas on all other plates, only small amounts of CPA, ranging between 10 µg/kg and 90 µg/kg, were measured.

Among the samples taken from the biofilm cocultures of *Penicillium glabrum* and *Penicillium camemberti*, the highest CPA level of 300 µg/kg was measured after incubation on lactose agar at 25 °C, and the lowest level of 40 µg/kg was measured after incubation on starch/glucose at 25 °C. The mycotoxins in the extracts of the samples, which were incubated at 4 °C, were below the limit of detection, except for the samples taken from PDA.

The a_w_ values of the agar types were 0.95 ± 0.00 for PDA, 0.93 ± 0.03 for starch/glucose agar, 0.95 ± 0.01 for starch agar, 0.95 ± 0.00 for lactose agar, and 0.94 ± 0.00 for galactose/glucose agar, which were all comparable to the a_w_ value of 0.94 ± 0.00 for cheese. The low variation indicated that water activity had no influence on the variation in the results. Therefore, incubation at lower temperatures reduces the growth of mold and, as a consequence, the production of mycotoxins.

### 3.4. Low Storage Temperatures Induce Mycotoxin Production in Inoculated Purchased Cheese Samples

Two loaves of Camembert cheese with a pH of 6.8 and an a_w_ value of 0.94 were cut into four quarters and divided horizontally to inoculate them with spores of *Penicillium chrysogenum*, *Mucor hiemalis*, and *Penicillium glabrum*. The inoculated samples were incubated at 4 °C and 25 °C to monitor mycotoxin production.

Two untreated aliquots contained mycotoxins maximally below the limit of detection, whereas one contained 20 µg/kg CPA at the start of the experiment. It was further tested whether subsequent incubation at 4 °C or 25 °C could induce mycotoxin production. For the 4 °C reference sample, a concentration of 90 µg/kg CPA was detected in the crust after 14 days, and 10 µg/kg CPA was detected in the inner part of the sample stored at 25 °C. In the other reference samples, no mycotoxins were detected.

The contaminated cheese samples revealed that CPA production was increased, especially in the crust where *Penicillium camemberti* was located, and that temperature did not necessarily reduce mycotoxin production (Table 4). For *Penicillium glabrum* contamination, the CPA level in the cold-stored samples doubled compared with those in the samples stored at 25 °C. Approximately three-fold greater levels of CPA were measured in cheese samples inoculated with *Penicillium chrysogenum* stored at 4 °C than in samples stored at 25 °C. In contrast, *Mucor hiemalis* did not induce CPA production at 4 °C, but high levels of CPA were measured in the samples stored at 25 °C.

## 4. Discussion

This study examines the effects of fungal–fungal cocultures, which lead to a shift in secondary metabolite production [15]. The key questions are whether these metabolic alterations adversely affect edible mold species, such as *Penicillium camemberti*, and whether an excess of *Penicillium camemberti* potentially acts as a protective layer for cheese.

The growth behavior of *Penicillium camemberti* and its ability to produce toxins is strain dependent. For example, Le Bars reported high variability in the CPA production of 20 different strains of *Penicillium camemberti* [16]. Furthermore, Casquete et al. [17] used *Penicillium camemberti* CBS273, which is a strain of unknown origin obtained from dust, which showed little CPA production, except at an incubation temperature of 25 °C and an a_w_ value of 0.95. The strain used in this work was isolated before March 1986 from pork meat and showed low CPA production at 4 °C and high production at 25 °C.

Furthermore, the synthesis of secondary metabolites is highly dependent on environmental factors such as a change in the pH value, the water activity of the growth medium or the carbon or nitrogen source available in the medium [18]. Specific pathways can be switched on or off due to such environmental signals [19]. This observation was extended in this work to biofilm experiments involving two different types of agars. The monosaccharide agar promoted mycotoxin production more than the lactose agar did. Unexpectedly, the coexpression of lactic acid, which caused no mycotoxin-dependent color change, induced the highest CPA yield in the biofilm experiments with both monosaccharide and lactose agents. Thus, the experiments also revealed that relying on visual inspection of food in the absence of mycotoxins is not sufficient. The observed dependency of the production of secondary metabolites on environmental factors is in line with a previous publication showing that mold growth and toxin production in food are influenced by temperature, water content, and species strain [9].

The present results showed that incubation with *Penicillium camemberti* biofilms at lower temperatures reduced the growth of mold and therefore the production of mycotoxins. This finding is in line with a previous report on CPA production by *Penicillium camemberti*, in which CPA production was lower at refrigerator temperatures and higher at room temperature [16].

The cocultures showed that it is relevant which mold species compete with *Penicillium camemberti* for resources and that mycotoxin production depends on the species involved.

In this study, the *Penicillium camemberti* strain grown on purchased cheese was able to produce CPA. The amount of CPA varied not only depending on the storage temperature but also on the type of cocultured strain. Cocultures with *Penicillium* species induced the production of the highest amount of CPA. However, *Mucor hiemalis* induced CPA production only at 25 °C but not at 4 °C. These findings clearly indicate that further investigations on fermented cheese are needed to understand the regulatory mechanisms that cause these observed differences. To answer the questions posed at the beginning of the discussion, it can be summarized that *Penicillium camemberti* was indeed influenced by contaminating strains, as CPA production changed in the coculture experiments. Furthermore, in this study *Penicillium camemberti* did not form a protective layer that inhibited the fungal growth and mycotoxin production of *Penicillium camemberti* or other species.

The aim of this study was to determine the effectiveness of *Penicillium camemberti* in preventing the invasion of contaminating molds in Camembert cheese. This effect was only observed to a limited extent. In particular, the parallel cultures showed a strong increase in mycotoxin production except for *Paecilomyces variotii*, which confirms the EFSA conclusion that *Penicillium camemberti* does not qualify for a fully qualified presumption of safety assessment.

## 5. Conclusions

The aim of the study was to determine the ability of a *Penicillium camemberti* layer on Camembert cheese to prevent the growth of contaminating mold. This hypothesis could only be confirmed to a limited extent, based on the analysis of a single cheese batch. Our results show that even small and imperceptible patches of mold can produce significant quantities of mycotoxins. However, the extent depends on the species. Consequently, it is advisable, particularly for foods with high water content like cheese, to entirely discard any products that show signs of mold. It is important to note that foods have different water contents, different sugar contents, different storage temperatures, and other parameters that may affect mycotoxin production. Therefore, further studies addressing mold contaminations in putatively safe foodstuffs are required to strengthen food safety.

## Figures and Tables

**Table 1:** Increases in the measured levels of mycotoxins in parallel cocultures on PDA compared with those in pure cultures.

Parallel Cocultures	Mycotoxin Production Compared to Pure Cultures			
	CPA	Chaetoglobosin A	Chaetoglobosin C	Chaetoglobosin F
*C. globosum*		107%	115%	210%
*P. camemberti*	912%			
	CPA	Fumonisin B_2_	Fumonisin B_4_	Fumonisin B_6_
*A. niger*		24%	630%	310%
*P. camemberti*	none			
	CPA	Viriditoxin		
*P. variotii*		none		
*P. camemberti*	none			
	CPA	Citrinin	Radicinol	
*P. citrinum*		104%	59%	
*P. camemberti*	279%			
	CPA	Roquefortine C	Roquefortine D	
*P. chrysogenum*		211%	79%	
*P. camemberti*	71%			

**Table 2:** Biofilm cultivation of *Penicillium camemberti* with contaminating fungi or with lactic acid bacteria on lactose agar and on galactose/glucose agar (<LOD, under limit of detection). The samples were measured once per fungal contamination and per agar.

Agar Type	Microorganisms	Mycotoxins			
		Citrinin	CPA	Radicinol	Viriditoxin
Lactose agar	*A. niger*	<LOD	<LOD	<LOD	<LOD
	*C. globosum*	<LOD	<LOD	<LOD	<LOD
	*P. chrysogenum*	<LOD	<LOD	<LOD	<LOD
	*P. citrinum*	20	150	<LOD	<LOD
	*P. variotii*	<LOD	<LOD	<LOD	5
	Lactic acid bacteria	<LOD	1000	<LOD	<LOD
	No contaminating mold	<LOD	<LOD	<LOD	<LOD
Monosaccharide agar (galactose/glucose)	*A. niger*	<LOD	60	<LOD	<LOD
	*C. globosum*	<LOD	580	<LOD	<LOD
	*P. chrysogenum*	<LOD	50	<LOD	<LOD
	*P. citrinum*	400	5	1810	<LOD
	*P. variotii*	<LOD	3	<LOD	170
	Lactic acid bacteria	<LOD	719	<LOD	<LOD
	No contaminating mold	<LOD	<LOD	<LOD	<LOD
					in µg/kg

LODs: citrinin (0.11 µg/kg), CPA (4 µg/kg), radicinol (0.66 µg/kg), viriditoxin (2.5 µg/kg).

**Table 3:** Contaminants *Penicillium chrysogenum*, *Mucor hiemalis,* and *Penicillium glabrum* with *Penicillium camemberti* biofilms cocultivated at 4 °C and 25 °C (<LOD, under the limit of detection).

Species	Agar Type	Mycotoxins and Respective Incubation Temperature					
		CPA		Roquefortine C		Roquefortine D	
		4 °C	25 °C	4 °C	25 °C	4 °C	25 °C
*Penicillium chrysogenum*	PDA	<LOD	7090	<LOD	140	<LOD	10
	Starch/glucose	<LOD	60	<LOD	<LOD	<LOD	<LOD
	Starch	40	540	<LOD	<LOD	<LOD	<LOD
	Lactose	20	<LOD	<LOD	<LOD	<LOD	<LOD
	Galactose/glucose	50	<LOD	<LOD	<LOD	<LOD	<LOD
*Mucor hiemalis*	PDA	10	2460	<LOD	<LOD	<LOD	<LOD
	Starch/glucose	20	<LOD	<LOD	<LOD	<LOD	<LOD
	Starch	10	<LOD	<LOD	<LOD	<LOD	<LOD
	Lactose	20	<LOD	<LOD	<LOD	<LOD	<LOD
	Galactose/glucose	<LOD	90	<LOD	<LOD	<LOD	<LOD
*Penicillium glabrum*	PDA	50	70	<LOD	<LOD	<LOD	<LOD
	Starch/glucose	<LOD	40	<LOD	<LOD	<LOD	<LOD
	Starch	<LOD	80	<LOD	<LOD	<LOD	<LOD
	Lactose	<LOD	300	<LOD	<LOD	<LOD	<LOD
	Galactose/glucose	<LOD	90	<LOD	<LOD	<LOD	<LOD
							in µg/kg

LODs: CPA (4 µg/kg), roquefortine C/D (0.06/0.5 µg/kg).

**Table 4:** Levels of mycotoxins measured in camembert cheese samples contaminated with *Penicillium chrysogenum*, *Mucor hiemalis*, and *Penicillium glabrum* stored at 4 °C and 25 °C for 14 days. The samples were measured once for each temperature and each fungal contamination.

Species	Cheese Spot	Mycotoxins and Respective Incubation Temperature			
		CPA		Roquefortine C	
		4 °C	25 °C	4 °C	25 °C
*Penicillium chrysogenum*	Crust	530	150	10	10
	Inner core	80	<LOD	<LOD	2
*Mucor hiemalis*	Crust	<LOD	440	<LOD	<LOD
	Inner core	<LOD	50	<LOD	<LOD
*Penicillium glabrum*	Crust	420	190	<LOD	<LOD
	Inner core	<LOD	80	<LOD	<LOD
					in µg/kg

LODs: CPA (4 µg/kg), roquefortine C (0.06 µg/kg).

## Data Availability

The original contributions presented in the study are included in the article, further inquiries can be directed to the corresponding author.
